# Detection and quantification of adulteration in milk and dairy products: A novel and sensitive qPCR-based method

**DOI:** 10.1016/j.fochms.2022.100074

**Published:** 2022-01-10

**Authors:** Rodrigo Giglioti, Hiago Polli, Bianca Tainá Azevedo, Luciana Morita Katiki, Anibal Eugênio Vercesi Filho

**Affiliations:** Instituto de Zootecnia, Rua Heitor Penteado, n. 56, Nova Odessa, São Paulo 13380-011, Brazil

**Keywords:** Adulteration, Detection, Quantification, DNA, Milk, Dairy products

## Abstract

Species identification in dairy products has a notable importance in food traceability and adulteration control and consequently has a significant effect on the final economic value of foods. In the present study, we developed a method based on real-time quantitative PCR (qPCR) for detection and quantification of cow DNA in DNA samples from milk and dairy products from buffaloes, goats, and sheep. The qPCR reactions showed high specificity, and the amplifications only occurred to species-specific primers. The calibration curves allowed for the quantification of the amount of DNA of each species-specific primer, and the established detection limit was 0.016 ng for the four species. The detection limit of cow DNA in buffalo, goat and sheep DNA samples was 0.1% (0.01 ng). Although the present study aimed to detect and quantify cow DNA in buffalo, goat, and sheep dairy products, we believe that the qPCR assays can also be directed to differentiate and quantify goat × sheep, and/or buffalo × goat/sheep.

## Highlights

• A novel qPCR method was able to detect and quantify cow, buffalo, goat and sheep DNA in milk and dairy products.
• Established detection limit was 0.016 ng for the four species.
• The limit of detection of cow DNA in buffalo, goat and sheep DNA samples was 0.1% (0.01 ng)
• This method is able to detect and quantify adulteration between cows, buffaloes, goats and sheep dairy samples.

## 1 Introduction

Milk, milk-based products, and milk derivatives represent an important group of food commodities, with high nutritional value and wide consumption by a large segment of consumers (Cossignani, Pollini, & Blasi, 2019). One of the most common problems encountered in the marketing of dairy products is the replacement of milk by dairy products of lower commercial value, due to price differences and seasonal availability making this attractive to farmers and producers (Di Domenico, Di Giuseppe, Rodríguez, & Cammà, 2016). Most of these unreported substitutions are performed by adding cow's milk to buffalo, sheep, and goat dairy products. However, these adulterations not only occur by adding cow's milk to these products. For instance, the replacement of sheep milk by goat milk in dairy products is a common problem because sheep milk has a higher price (Yangilar, 2013). In addition, there are mixed herd of goats and sheep that can results in accidental or fraudulent replacement of sheep milk by goat milk and vice-versa (Pappas et al., 2008). According to Di Domenico et al. (2016), unintentional substitutions can also occur when multiple species are handled on the same manufacturing equipment.

Furthermore, species identification of dairy products has a great importance due to frequent human adverse reactions (allergies) to some milk proteins. It also allows for the detection of adulteration in the form of the substitution of a less costly type of milk for one of a higher quality (Bottero et al., 2003, Bottero et al., 2002). According to Haenlein (2004), goat milk is particularly suitable for people with cow milk allergies. In contrast, the consumption of milk or dairy products from goats contaminated with cow milk can cause allergic processes in individuals potentially allergic to cow milk. Due to the increasing consumption of buffalo milk derivatives, seasonality, and for presenting greater added value when compared to dairy products from cows, the addition of variable amounts of cow milk during the manufacturing of buffalo dairy products may occur, which constitutes fraud by product adulteration (Azevedo et al., 2021). Food authentication is a rapidly growing field because of increasing consumer awareness regarding food quality and safety (Cossignani et al., 2019). Consequently, the authenticity of dairy products often has a strong effect on the final economic value of the food (Di Stefano et al., 2012).

PCR-based methods for the detection and differentiation of species have usually been applied due to their high specificity, sensitivity, and speed. Their use on commercial dairy products is achievable, owing to the presence of recoverable DNA derived from somatic cells (leukocytes and breast cells), even after heat treatment (Lipkin, Shalon, Khatib, Soller, & Friedmann, 1993). The somatic cells levels in the milk may be influenced by several factors, such as animal species, milk production level, lactation stage, and individual and environmental factors, as well as management practices (Li, Richoux, Boutinaud, Martin, & Gagnaire, 2014). According to Azevedo et al. (2021), owing to the high variation in the amount of extracted DNA from somatic cells in the milk samples and its derivatives, highly sensitive DNA extraction methods and molecular genetic methodologies are required. The PCR methods using mitochondrial DNA as a specific target for detecting cow DNA in dairy products from buffalos, goats, and sheep have been frequently studied (Bottero et al., 2002, Bottero et al., 2003, Mafra et al., 2004, Feligini et al., 2005, Lopparelli et al., 2007, Cottenet et al., 2011; Dalmasso, Civela, La Neve & Bottero, 2011; Gonçalves et al., 2012, Di Domenico et al., 2016, Liao et al., 2017). However, most studies were developed to detect only the presence of adulteration and not to quantify the amount of contamination. Furthermore, amplifications of different regions of species-specific DNA fragments based on end-point PCR with subsequent agarose gel electrophoresis does not provide any quantification of the targets (Agrimonti, Pirondini, Marmiroli, & Marmiroli, 2015). For this, the use of quantitative PCR (qPCR) is recommended. In addition to establishing a detection limit, it also calculates the amount of contaminating DNA. Thereby, the present study developed a qPCR to simultaneously detect and quantify the presence of cow DNA in dairy products from buffaloes, goats, and sheep.

## 2 Material and methods

### 2.1 Experimental samples and DNA extraction

All DNA samples evaluated in this study are provided from the DNA collection of the Biotechnology Laboratory of the Instituto de Zootecnia-IZ, Nova Odessa, São Paulo state, Brazil. Twelve milk samples (3 cow, 3 buffalo, 3 goat, and 3 sheep) were submitted to DNA extractions according to the method by Reale, Campanella, Merigioli, and Pilla (2008), following the modifications recommended by Giglioti et al. (2020). DNA samples from dairy products were extracted according to the method described by Azevedo et al. (2021). The samples of dairy products (different types of cheeses – data not shown) included: 22 buffalo samples, 3 goat samples, and 3 sheep samples. All DNA samples were diluted to a final concentration of 5 ng/µL.

### 2.2 PCR and DNA sequencing

**Fig. 1:**
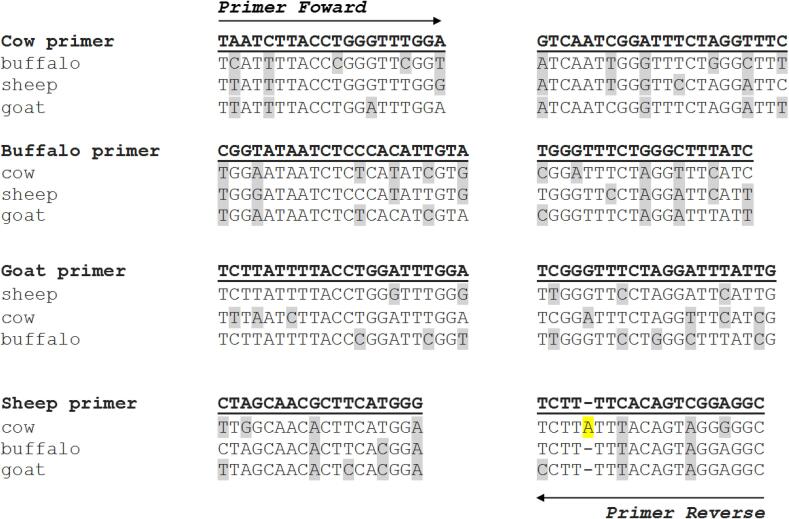
Specific primer alignments of mitochondrial DNA (*mt*DNA). Each specific primer was aligned with the respective non-specific species. Gray markings represent the differences between the specific primer with the same region of the non-specific species. The yellow marking (cow) represents an insertion compared to the other species. (For interpretation of the references to colour in this figure legend, the reader is referred to the web version of this article.)

A set of specific primers were designed using the sequences flanking the cytochrome *c* oxidase subunit 1 mitochondrial gene (cox1DNA) for each species (Supplementary material Table 1). DNA extracted from milk samples (cow, buffalo, goat, and sheep, n = 12) were subjected to PCR reactions. The assays were performed for a final volume of 50 µL, using 0.2 µL Platinum™ Taq DNA Polymerase High Fidelity (5 U/µL; Invitrogen, Carlsbad, US), 1.5 µL 50 mM MgSO_4_ (Invitrogen), 5 µL 10 × Taq DNA Polymerase PCR buffer [(600 mM Tris-SO_4_ (pH 8.9), 180 mM (NH_4_)_2_SO_4_; Invitrogen)], 1 µL 10 mM dNTP mix (Sigma-Aldrich, St. Louis, MO, USA), 2 µL of each 10 μM forward and reverse primers (Supplementary material Table 1), and 2 µL DNA (10 ng). The PCR amplification conditions were: initial denaturation at 95 °C for 5 min, followed by 35 cycles at 95 °C for 30 s (denaturation), 55 °C for 45 s (annealing), and 68 °C for 1 min (extension), with a final extension at 68 °C for 10 min. The amplification products were submitted to electrophoresis in 2.0% agarose gel, stained with ethidium bromide, and visualized under ultraviolet light. The PCR products were purified using a PureLink® PCR purification kit (Invitrogen) according to the manufacturer's recommendations. The sequencing reaction was performed using a BigDye Terminator v3.1 cycle sequencing kit (Applied Biosystems, Foster City, CA, USA) then analysed with an ABI Prism 3730XL DNA analyser (Applied Biosystems). The DNA sequences obtained were aligned using CLUSTAL/W software (Thompson, Higgins, & Gibson, 1994) and compared with those already deposited in GenBank. The contig sequences were also evaluated by BLAST (http://blast.ncbi.nlm.nih.gov/Blast.cgi). One nucleotide sequence from each species evaluated in this study was deposited in GenBank (MZ668303, MZ668304, MZ782619, and MZ782720). The Fig. 1 shows the alignments of species-specific qPCR primers.

### 2.3 qPCR reactions

A set of qPCR specific primers were designed using the regions of the *cox1*DNA gene sequenced delimited by the primers designed for conventional PCR (Supplementary material Table 1). The qPCR assay was performed in 10 μL reaction volumes using a Rotor-Gene Q thermocycler (Qiagen, Venlo, Netherlands). Each reaction contained 5.4 μL of sterile water, 2 μL of 5 X HOT FIREPol EvaGreen® HRM mix (Solis Biodyne, Tartu, Estonia), 0.3 µL of each primer (10 μM), and 2 μL of DNA (10 ng). A negative template control was included in each PCR run. The qPCR was performed using the following conditions: initial denaturation at 95 °C for 12 min, followed by 35 cycles of denaturation (95 °C for 15 s), annealing (63 °C for 20 s), and extension (72 °C for 20 s). After amplification, HRM (high resolution melting) analysis was performed during dissociation curves from 70 to 92 °C in 0.2 °C increments, rising at 0.1 °C/2 s. PCR runs were performed jointly according to primer-specific comparisons between cow with the other three species (cow × buffalo, cow × goat, and cow × sheep). The standard samples (cow, buffalo, goat, and sheep) used for each PCR reaction were those previously sequenced (Section 2.2).

### 2.4 Specificity, sensitivity, and DNA quantification

The specificity of each specific-primer was checked for the presence or absence of non-specific amplifications by the melting peak temperature (°C) and changes in the shape of curves normalized by HRM analysis. The analytical sensitivity was evaluated using serial 5-fold dilutions (5^1^ to 5^−5^, 10 ng to 0.0032 ng) from DNA sample controls from each species (specific-species samples sequenced). To estimate the DNA quantity of each target species, calibration curves were standardized from serial 5-fold dilutions (described above), and the quantification ranges were determined for each species. Reaction efficiency (E), slope, and coefficient of determination (r^2^) were also determined for each species. Furthermore, the analytical sensitivity was also evaluated by testing decreasing concentrations of cow DNA in buffalo, goat and sheep DNA samples: 50% (5 ng), 10% (1 ng), 5% (0.5 ng), 2% (0.2 ng), 1% (0.1 ng), 0.5% (0.05 ng), and 0.1% (0.01 ng). The limit of detection was set at last dilution which presented ≥ 90% of detection, and for each concentration 10 technical replicates were used.

### 2.5 Detection of cow DNA in sheep, goat, and buffalo dairy samples

The DNA samples of dairy products (22 buffalo samples, 3 goat samples, and 3 sheep samples) were evaluated by qPCR to detect and quantify the presence of cow DNA (section 2.3 and 2.4). The differences between the amount of DNA (ng) between cow and buffalo were transformed into percentages of cow DNA: (cow DNA – [cow DNA + buffalo DNA])*100.

## 3 Results

### 3.1 DNA sequencing

The amplicons from PCR reactions were specific for each specific primer and allowing for its use in sequencing reactions (Supplementary Fig. 1). The sequenced samples were deposited in GeneBank (access numbers: MZ668303, MZ668304, MZ782619, and MZ782720) and were used as controls for the qPCR reactions.

### 3.2 qPCR reactions

#### 3.2.1 Specificity, sensitivity, and DNA quantification

**Fig. 2:**
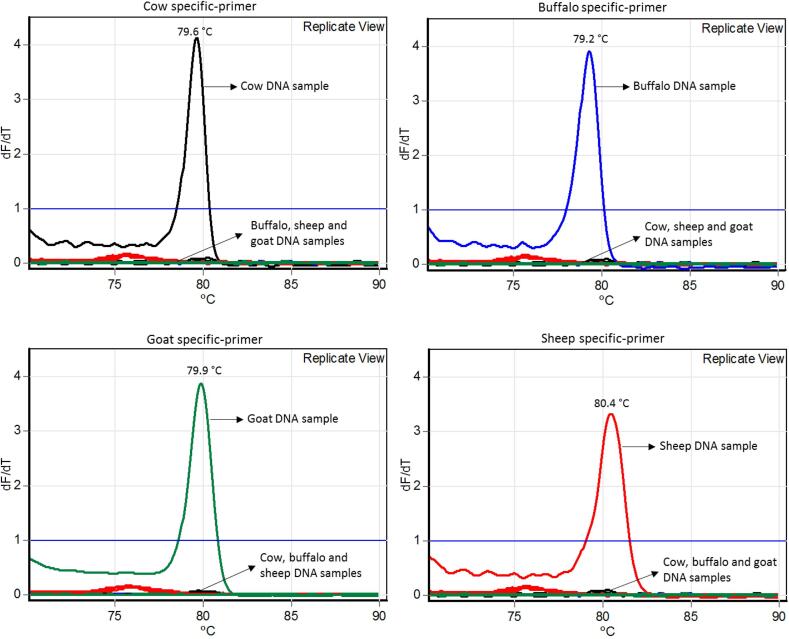
Temperature of melting peaks obtained using qPCR assay using specific primers. Black line: cow primers; blue line: buffalo primers; green line: goat primers; and red line: sheep primers. (For interpretation of the references to colour in this figure legend, the reader is referred to the web version of this article.)

The primer designed for each species was specific and did not present unspecific amplifications when contrasted to unspecific samples (cow × buffalo, cow × goat, and cow × sheep) (Fig. 1). Samples from buffaloes, goats, and sheep were also contrasted with each other and did not show non-specific amplifications. Each specific primer showed a specific peak melting temperature (Fig. 2).

**Fig. 3:**
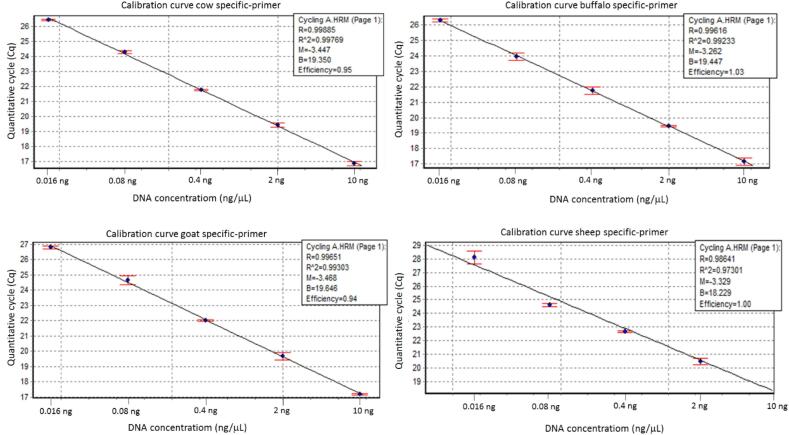
Amplification curves and reaction efficiencies from species-specific primers: cow (top left), buffalo (top right), goat (lower left), and sheep (lower right). The lines presented for each species represent the replicate view (ten technical replicates).

**Fig. 4:**
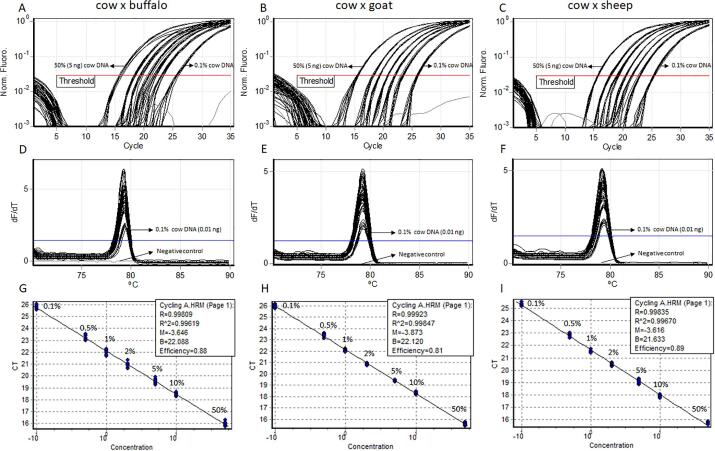
Amplification curves, temperature of melting peaks and reaction efficiencies from analytical sensitivity test of detection cow DNA in buffalo (A, D, and G), goat (B, E, and H) and sheep (C, F, and I) DNA samples, respectively. Concentrations evaluated in each test: 50% (5 ng), 10% (1 ng), 5% (0.5 ng), 2% (0.2 ng), 1% (0.1 ng), 0.5% (0.05 ng), and 0.1% (0.01 ng). For each concentration tested were used ten technical replicates.

The limit of detection using serial 5-fold dilutions for each species-specific analysis was the same, 0.016 ng (Fig. 3). Calibration curves using the serial dilutions of each specific sample allowed us to quantify and estimate the detection limits for each specific species. The reaction efficiency observed for cows, buffaloes, goats, and sheep were 95%, 103%, 94%, and 100%, respectively (Fig. 3). The limit of detection of cow DNA in buffalo, goat and sheep DNA samples was 0.1% (0.01 ng) (Fig. 4). For the two established limits of detection (0.016 ng and 0.1% (0.01 ng)), there were amplifications of the 10 technical replicates (100%) (Supplementary Table 2).

**Fig. 5:**
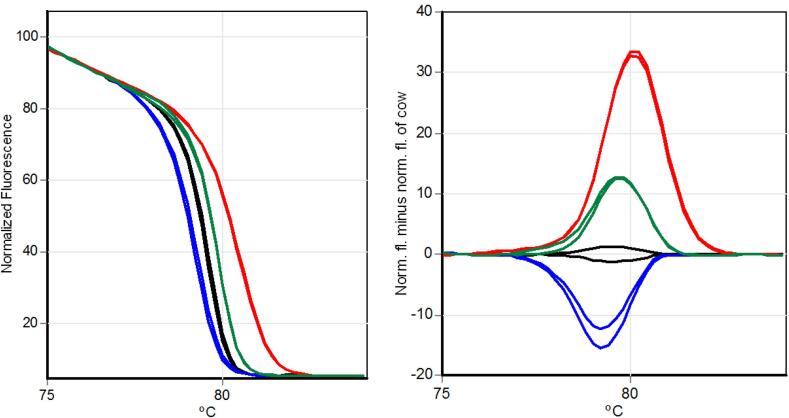
High-resolution melting (HRM) curve profiles of amplicons from mitochondrial primers using bovine (black line), buffalo (blue line), goat (green line), and sheep (red line) species. Normalized fluorescence signals (left) and difference plot (right): the results are presented as normalized fluorescence of sheep, goat, and buffalo samples minus normalized fluorescence of the bovine standard sample. (For interpretation of the references to colour in this figure legend, the reader is referred to the web version of this article.)

The differences between cow, buffalo, goat, and sheep were also verified by HRM analysis. Each species was accurately differentiated by the HRM plot curve (Fig. 5).

#### 3.2.2 Detection of cow DNA in sheep, goat, and buffalo dairy samples

Among sheep and goat cheese DNA samples, there was no verified presence of cow DNA. Regarding the buffalo dairy samples, cow DNA was detected and quantified in 12 samples (Table 1).

**Table 1:** Quantity (ng) of cow and buffalo DNA from buffalo cheese samples and percentage of cow DNA detected in each sample.

Sample	DNA quantity (ng)		% Cow DNA*
	Cow DNA (ng)	Buffalo DNA (ng)	
1	1.97	2.49	44
2	0.00	2.33	0
3	1.40	3.45	29
4	0.00	3.36	0
5	2.58	1.33	66
6	0.00	2.92	0
7	0.91	4.05	18
8	1.59	1.75	48
9	0.00	3.87	0
10	0.09	3.49	2
11	0.00	2.28	0
12	0.21	4.06	5
13	0.00	2.98	0
14	1.83	0.58	76
15	1.76	2.40	42
16	0.00	2.61	0
17	0.31	3.49	8
18	0.04	1.46	3
19	0.00	2.95	0
20	0.00	2.96	0
21	0.00	2.58	0
22	0.79	0.31	72

*=% Cow DNA: (cow DNA – [cow DNA + buffalo DNA])*100.

## 4 Discussion

Food adulteration commonly involves economically-motivated adulteration, with unconscionable producers aiming to raise profit margins using any resource necessary without giving any consideration to consumer safety (McGrath et al., 2018, Moore et al., 2012). Dairy products are a group of food that play an important role in feeding the population. Milk is a relatively costly raw material and from an economic aspect, can be an attractive product for modifications by partial replacement with other dairy and non-dairy ingredients (De La Fuente and Juaréz, 2005). The adulteration caused by dairy adulteration is a problem acknowledged by the authorities; hence, the developing of effective methods for the detection of falsified and/or adulterated products is essential (Hanganu & Chira, 2021). Thus, the development of analytical techniques with high specificity and sensitivity for adulteration detection in dairy products are welcome. The quantitative PCR assays developed here allowed for the detection and quantification of cow *cox1*DNA in dairy samples from sheep, goats, and buffaloes. According to Rodrigues, Morelli, and Jansen (2017), the use of the cytochrome *c* oxidase subunit 1 mitochondrial gene is highly efficient for discriminating vertebrate and invertebrate species. In addition to detecting cow DNA in the other species samples, the present methodology can be applied to differentiate between and quantify buffalo, goat, and sheep DNA samples. The adulteration does not only occur with the addition of cow's milk to these products. For example, replacing sheep milk with goat milk in dairy products is a common problem, as sheep milk has a higher price (Geller et al., 2013, Yangilar, 2013).

Several studies have been performed emphasizing the detection of contamination of non-cow dairy products by the presence of cow milk. However, most of this research has only established the detection limit of cow DNA without quantifying the level of contaminating DNA (Bottero et al., 2002, Bottero et al., 2003, Dalmasso et al., 2011, Di Domenico et al., 2016, Di Pinto et al., 2004, Feligini et al., 2005, Golinelli et al., 2014, Gonçalves et al., 2012, Reale et al., 2008, Rodrigues et al., 2012, Zhang et al., 2007). The cow DNA contamination during the processing of dairy products can occur intentionally or unintentionally. Thus, conclusive evidence of the occurrence of adulteration requires, in addition to detection, the quantification of food components (Zhang et al., 2007). The present study allowed quantification and comparison of the DNA concentrations between species, allowing us to estimate the amount of contamination of the cow species. The initial 10 ng concentration for each species allowed the construction of calibration curves, whose detection limit was 0.016 ng for each species. In addition, the detection limit established in the cow DNA detection in buffaloes, goats and sheep DNA samples was 0.1%.

In the literature, there are few studies that used calibration curves (absolute quantification) to estimate the level of cow DNA contamination in other dairy products. Mafra et al. (2004) described a simple duplex PCR method to identify cow and ovine species in cheeses and quantify cow milk in ovine cheeses using a normalized calculation obtained by the ratio of the band intensities from PCR products. The quantification of contamination based on the intensity of the bands of the conventional PCR products can present lower efficiency, since the evaluations by intensity bands of the PCR products can be carried out subjectively. In the present study, the calibration curves used to detect and quantify the DNA of each species were based on quantitative cycle values (Cq), which provide a more accurate estimate of the absolute amount of DNA. Liao et al. (2016) developed a qualitative and quantitative PCR for adulteration identification of cow milk in commercial goat milk powders. However, in this study, specific primers for the detection and quantification of sheep DNA samples were not designed. We suggest that to estimate the precise adulteration amount, DNA quantification of the contaminating species and the species evaluated should be performed simultaneously to determine the proportion of contaminating DNA in relation to the evaluated DNA sample.

The EvaGreen intercalating dye was determined to be highly specific for differentiating between cows, sheep, goats, and buffaloes. This is the first report that differentiated and quantified DNA samples of cow, sheep, goat, and buffalo using intercalant dye EvaGreen. One of the reasons that intercalant dye is often used is that it is relatively inexpensive compared with other detection chemistries. Agrimonti et al. (2015) developed a quadruplex PCR assay for detecting and quantifying adulteration in dairy products using intercalant dye SYBR Green. In this study, the quadruplex PCR failed to detect goat and sheep milk in some cheeses, but they were detected in singleplex PCR reactions. In the present study, we used singleplex PCR reactions in order to increase the sensitivity and specificity of the reactions, in addition to providing the construction of calibration curves for the quantification of species DNA with high accuracy. Furthermore, some problems inherent to the use of SYBR Green dye have been reported, such as inhibition of the PCR assay, preferential binding to GC-rich sequences, and effects on melting curve analysis (Gudnason, Dufva, Bang, & Wolff, 2007). EvaGreen is a saturated dye that intercalates in all single nucleotides of the double-stranded DNA. It displays relatively low PCR inhibition and relatively low tendency to cause nonspecific amplification of the dye. It also ensures high specificity, sensitivity, and stability of assays and melt peaks of different amplicons, which could be obviously identified using melting curve analysis (Cheng et al., 2013, Khan et al., 2011, Mao et al., 2007). In addition, the use of the EvaGreen intercalating saturating dye also enabled the differentiation between the different species evaluated by the HRM method. Although, the qPCR assays using specific primers were performed in separate tubes, differentiation by HRM analysis was possible, and different shapes of the normalized curves obtained were observed between the four species.

Based on the results obtained in the present study, the detection and quantification of adulteration by adding cow’s milk into milk or dairy products from goats, sheep, or buffaloes can be performed following two steps: (i) detection of cow DNA and/or (ii) quantification of cow DNA in the sample (only performed when the presence of cow DNA is detected). The quantification of contamination can be an essential factor for raising the hypothesis of why the adulteration occurred, since variable amounts of cow DNA in the samples can be associated with intentional and unintentional contaminations. According to Dalmasso et. al (2011), detecting very small amounts of cow's milk in dairy products can be a disadvantage, as it is difficult to distinguish adulteration from unintentional contamination occurring during processing. Thus, it is necessary that a regulatory control agency can inspect and establish some detection and/or quantification limit of adulteration. Quantitative results in adulteration control should be understood as approximate values, and the authentication of cheeses with mixed milk remains a challenge for food analysts (Mayer, Bürger, & Kaar, 2012). The present methodology is not limited only to the detection and quantification of cow DNA in samples of sheep, goats, and buffaloes but also to the detection and quantification of DNA among these three species when required. Further, we also suggest that this method can be also standardized and applied to other types of food in addition to milk and dairy products.

## CRediT authorship contribution statement

**Rodrigo Giglioti:** Conceptualization, Data curation, Formal analysis, Investigation, Methodology, Project administration, Software, Supervision, Validation, Visualization, Writing – original draft, Writing – review & editing. **Hiago Polli:** Conceptualization, Supervision, Validation, Visualization, Writing – original draft, Writing – review & editing. **Bianca Tainá Azevedo:** Conceptualization, Supervision, Validation, Visualization, Writing – original draft, Writing – review & editing. **Luciana Morita Katiki:** Conceptualization, Supervision, Validation, Visualization, Writing – original draft, Writing – review & editing. **Anibal Eugênio Vercesi Filho:** Funding acquisition, Investigation, Project administration, Resources, Supervision, Validation, Visualization, Writing – original draft, Writing – review & editing.

## Declaration of Competing Interests

The authors declare that they have no known competing financial interests or personal relationships that could have appeared to influence the work reported in this paper.
