# Supplementary material for: Detection and quantification of adulteration in milk and dairy products: A novel and sensitive qPCR-based method
Source: Food Chem (Oxf). 2022 Jan 10;4:100074. doi: 10.1016/j.fochms.2022.100074 (PMC8991746; doi:10.1016/j.fochms.2022.100074)
Supplement: Supplementary data 1 [file mmc1.docx]

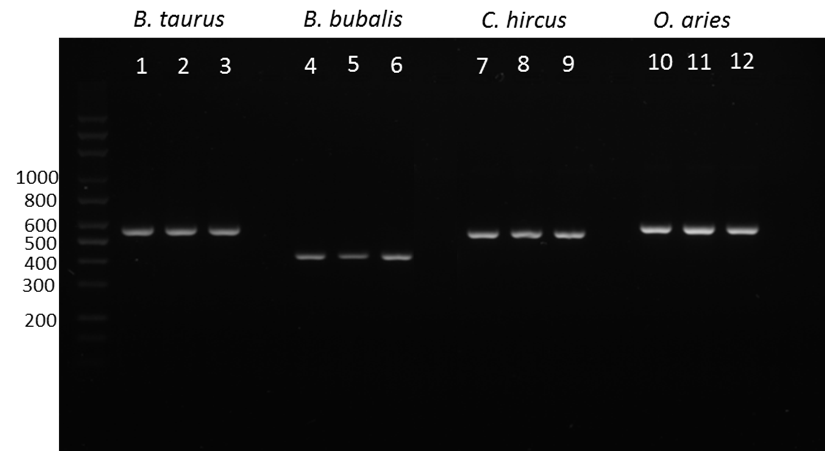


**Supplementary Fig. 1.** Photographs obtained of agarose gel electrophoresis of PCR products from mitochondrial genes (*mt*DNA) of a cow (1–3, 553 pb), buffalo (4–5, 415 pb), goat (7–9, 535 pb), and sheep (10–12, 572 pb).
