# Supplementary material for: Detection and quantification of adulteration in milk and dairy products: A novel and sensitive qPCR-based method
Source: Food Chem (Oxf). 2022 Jan 10;4:100074. doi: 10.1016/j.fochms.2022.100074 (PMC8991746; doi:10.1016/j.fochms.2022.100074)
Supplement: Supplementary data 2 [file mmc2.docx]

**Table 1.** Sequences of primers used in PCR and qPCR assays

| Assay | species | Primer | Sequence (5´–3´) | Product size (bp) |
| --- | --- | --- | --- | --- |
| PCR sequencing | *B. taurus* | *mt-PCR-bov-F* | F: CTACTCTCGCTCCCTGTAT | 553 |
|  |  | *mt-PCR-bov-R* | R: CATAGTGGAAATGTGCGACAA |  |
|  | *B. bubalis* | *mt-PCR-bub-F* | F: GCCGTGCTATTACTCCTTTCA | 415 |
|  |  | *mt-PCR-bub-R* | R: CTCCGTGAAGTGTTGCTAGT |  |
|  | *C. hircus* | *mt-PCR-goat-F* | F: CCTAGCAGGCATCTCTTCAAT | 535 |
|  |  | *mt-PCR-goat-R* | R: TCCGTGGAGTGTTGCTAATC |  |
|  | *O. aries* | *mt-PCR-goat-F* | F: CACCTGCGATGTCACAGTATC | 572 |
|  |  | *mt-PCR-goat-R* | R: AAGGGAGGAGTTGGCTAGAA |  |
|  |  |  |  |  |
|  | *B. taurus* | *mt-qPCR-bov-F* | F: TTAATCTTACCTGGGTTTGGA | 120 |
| qPCR |  | *mt-qPCR-bov-R* | R: GAAACCTAGAAATCCGATTGAC |  |
|  | *B. bubalis* | *mt-qPCR-bub-F* | F: CGGTATAATCTCCCACATTGTA | 106 |
|  |  | *mt-qPCR-bub-R* | R: GATAAAGCCCAGAAACCCA |  |
|  | *C. hircus* | *mt-qPCR-goat-F* | F: CTTATTTTACCTGGATTTGGA | 124 |
|  |  | *mt-qPCR-goat-R* | R: CAATAAATCCTAGAAACCCGA |  |
|  | *O. aries* | *mt-qPCR-goat-F* | F: CTAGCAACGCTTCATGGG | 87 |
|  |  | *mt-qPCR-goat-R* | R: GCCTCCGACTGTGAAAAGA |  |
