# Supplementary material for: Detection and quantification of adulteration in milk and dairy products: A novel and sensitive qPCR-based method
Source: Food Chem (Oxf). 2022 Jan 10;4:100074. doi: 10.1016/j.fochms.2022.100074 (PMC8991746; doi:10.1016/j.fochms.2022.100074)
Supplement: Supplementary data 3 [file mmc3.docx]

**Supplementary Table 2**. Quantification cycles (Cq) values from the analytical sensitivity for cow DNA detection in buffalo, goat and sheep DNA samples. Concentrations evaluated in each test: 50% (5 ng), 10% (1 ng), 5% (0.5 ng), 2% (0.2 ng), 1% (0.1 ng), 0.5% (0.05 ng), and 0.1% (0.01 ng). For each concentration tested were used ten technical replicates.

|  | Cycle quantitative (Cq) values | | |
| --- | --- | --- | --- |
| % Cow DNA | Buffalo samples | Goat samples | Sheep samples |
| 50% | 15.73 | 15.61 | 15.65 |
| 50% | 15.85 | 15.76 | 15.70 |
| 50% | 15.86 | 15.51 | 15.65 |
| 50% | 15.88 | 15.40 | 15.63 |
| 50% | 15.89 | 15.46 | 15.82 |
| 50% | 15.89 | 15.69 | 15.75 |
| 50% | 15.92 | 15.60 | 15.76 |
| 50% | 15.97 | 15.65 | 15.67 |
| 50% | 16.08 | 15.50 | 15.58 |
| 50% | 16.29 | 15.43 | 15.58 |
| 10% | 18.27 | 18.46 | 17.78 |
| 10% | 18.28 | 18.34 | 17.82 |
| 10% | 18.34 | 18.39 | 17.75 |
| 10% | 18.39 | 18.33 | 17.90 |
| 10% | 18.43 | 18.28 | 17.71 |
| 10% | 18.51 | 18.33 | 18.04 |
| 10% | 18.52 | 18.14 | 17.96 |
| 10% | 18.56 | 18.14 | 17.92 |
| 10% | 18.58 | 18.35 | 18.03 |
| 10% | 18.65 | 18.33 | 17.70 |
| 5% | 19.26 | 19.48 | 18.86 |
| 5% | 19.34 | 19.32 | 18.92 |
| 5% | 19.42 | 19.43 | 19.22 |
| 5% | 19.47 | 19.28 | 19.01 |
| 5% | 19.58 | 19.38 | 19.12 |
| 5% | 19.58 | 19.38 | 19.11 |
| 5% | 19.64 | 19.28 | 18.98 |
| 5% | 19.69 | 19.37 | 19.12 |
| 5% | 19.72 | 19.47 | 19.32 |
| 5% | 19.89 | 19.44 | 18.83 |
| 2% | 20.62 | 20.93 | 20.52 |
| 2% | 20.64 | 20.90 | 20.49 |
| 2% | 20.65 | 20.84 | 20.58 |
| 2% | 20.83 | 20.93 | 20.43 |
| 2% | 20.85 | 20.76 | 20.33 |
| 2% | 20.86 | 20.77 | 20.31 |
| 2% | 20.91 | 20.91 | 20.42 |
| 2% | 21.01 | 20.76 | 20.55 |
| 2% | 21.07 | 20.88 | 20.41 |
| 2% | 21.32 | 20.73 | 20.27 |
| 1% | 21.67 | 22.04 | 21.51 |
| 1% | 21.68 | 22.13 | 21.58 |
| 1% | 21.79 | 22.14 | 21.70 |
| 1% | 21.93 | 22.21 | 21.70 |
| 1% | 21.98 | 21.92 | 21.48 |
| 1% | 22.08 | 22.00 | 21.52 |
| 1% | 22.23 | 22.24 | 21.71 |
| 1% | 22.25 | 21.96 | 21.37 |
| 1% | 22.26 | 21.97 | 21.45 |
| 1% | 22.27 | 22.03 | 21.48 |
| 0.5% | 22.99 | 23.30 | 22.77 |
| 0.5% | 23.08 | 23.35 | 22.59 |
| 0.5% | 23.09 | 23.37 | 22.87 |
| 0.5% | 23.17 | 23.54 | 22.95 |
| 0.5% | 23.18 | 23.11 | 22.81 |
| 0.5% | 23.22 | 23.46 | 22.93 |
| 0.5% | 23.38 | 23.34 | 22.96 |
| 0.5% | 23.43 | 23.40 | 23.03 |
| 0.5% | 23.47 | 23.51 | 22.99 |
| 0.5% | 23.53 | 23.59 | 22.96 |
| 0.1% | 25.58 | 26.14 | 25.22 |
| 0.1% | 25.60 | 26.17 | 25.35 |
| 0.1% | 25.69 | 25.78 | 25.39 |
| 0.1% | 25.69 | 25.89 | 25.15 |
| 0.1% | 25.78 | 26.15 | 25.23 |
| 0.1% | 25.80 | 25.94 | 25.33 |
| 0.1% | 25.84 | 26.07 | 25.24 |
| 0.1% | 25.85 | 26.01 | 25.18 |
| 0.1% | 25.91 | 25.94 | 25.53 |
| 0.1% | 26.06 | 25.94 | 25.35 |
| NTC | NA | NA | NA |
| NTC | NA | NA | NA |

NTC: No template control
